# Probiotics as treatment for food allergies among pediatric patients: a meta-analysis

**DOI:** 10.1186/s40413-018-0204-5

**Published:** 2018-11-06

**Authors:** Carol Stephanie C. Tan-Lim, Natasha Ann R. Esteban-Ipac

**Affiliations:** College of Medicine, University of the Philippines Manila, Paz Mendoza Hall, 547 Pedro Gil Street, Ermita, 1000 Manila, Philippines

**Keywords:** Food allergy, cow’s milk allergy, Probiotics, Pediatrics

## Abstract

**Background:**

The burden of disease of food allergy is increasing worldwide. The standard of management is allergen avoidance and symptomatic treatment. Probiotics have been proposed to be beneficial for treatment and prevention of food allergy.

**Objective:**

To determine the effectiveness of probiotic administration in treating food allergies among pediatric patients.

**Methods:**

A systematic search of electronic medical literature databases was conducted. Manual search of the reference lists and search for unpublished articles were also done. All randomized controlled trials available from inception until February 19, 2018 were retrieved. The primary outcome of interest was relief of allergic symptoms, while the secondary outcome of interest was inducement of tolerance. Two independent authors did the search, screening, appraisal, and data abstraction. Data analysis and synthesis were done using RevMan 5.3 software. Subgroup analysis was done based on the probiotic strains and time periods in measuring the outcome. Exclusion sensitivity analysis was also done.

**Results:**

Nine trials involving 895 pediatric patients with cow’s milk allergy (CMA) were included in the review. The primary outcome of interest, relief of symptoms, was measured using the scoring index for eczema. Pooled results from two studies showed larger reduction in the scoring index among patients given probiotics, but this effect was imprecise (MD -1.30, 95% CI -3.88, 1.28). For the secondary outcome of interest, pooled results from four studies showed benefit of probiotics in inducing tolerance, but again this result is imprecise with significant heterogeneity (RR 0.58, 95% CI 0.34, 1.00). Subgroup analysis per probiotic strain showed benefit of *Lactobacillus rhamnosus* GG in inducing tolerance based on two studies involving infants with suspected cow’s milk allergy (RR = 0.41, 95% CI 0.28 to 0.62). Another subgroup analysis showed a duration-dependent effect associated with probiotic usage, with inducement of tolerance noted after at least 2 years (RR = 0.44, 95% CI 0.29 to 0.67).

**Conclusion:**

Analysis of available evidence shows moderate certainty that the use of probiotics can relieve symptoms of children with cow’s milk allergy. The reduction in certainty is due to imprecise results. Moreover, there is low certainty that probiotics can induce tolerance among children with cow’s milk allergy, due to problems of imprecision and attrition bias. In the subgroup analysis, *Lactobacillus rhamnosus* GG administration likely results in inducing tolerance among infants with suspected cow’s milk allergy. Only studies on CMA were analyzed since no studies were found on probiotics as treatment for other types of food allergy among children.

**Electronic supplementary material:**

The online version of this article (10.1186/s40413-018-0204-5) contains supplementary material, which is available to authorized users.

## Background

Food allergy is defined as “an adverse health effect arising from a specific immune response that occurs reproducibly on exposure to a given food”. Food allergens are specific components of food recognized by the individuals’ immune system that result in characteristic allergic symptoms [1]. The most serious and potentially fatal allergic reaction is anaphylaxis. Other allergic reactions include gastrointestinal manifestations such as vomiting, feeding disorders, reflux, abdominal pain, dysphagia, diarrhea, growth failure, and bloody stools; cutaneous manifestations such as urticaria, angioedema, flushing, pruritus, and eczema; and respiratory manifestations such as wheezing, dyspnea, nasal congestion, sneezing, and rhinorrhea [2].

The prevalence of food allergy is increasing worldwide, with the global prevalence approaching 10% [1, 3]. The epidemiology of food allergy varies by age group and geographic location. Children have higher rates of food allergy compared to adults. Shellfish allergy is more common in Asian countries, while peanut allergy is more common in Western countries. Other frequent food allergens include cow’s milk, egg and wheat [4, 5].

Despite the high burden of disease and potential risk of fatal outcomes, there is still no cure for food allergies. The standard of management is allergen avoidance and symptomatic treatment. For patients with cow’s milk allergy (CMA), cow’s milk protein is eliminated from the diet through extensively hydrolyzed protein formula or amino acid-based formula. Complete elimination of the food allergen is often difficult due to its widespread use in processed food [2]. Definitive treatment for food allergy, including various types of immunotherapy, is still undergoing extensive research [6].

Probiotic administration has been proposed to be effective for treatment and prevention of food allergy. The joint Food and Agriculture Organization and World Health Organization Expert Consultation defined probiotics as “live microorganisms that, when administered in adequate amounts, confer a health benefit on the host” [7]. Probiotics have been hypothesized to cause activation of local macrophages, modulation of local and systemic IgA production, and alteration of the pro- and anti-inflammatory cytokine profile, leading to modulation of response to food antigens [8]. Commonly researched probiotic strains are *Lactobacillus rhamnosus, Lactobacillus reuteri*, *Bifidobacteria spp*, *Lactobacillus casei*, *Lactobacillus acidophilus*, *Bacillus coagulans, Escherichia coli* strain Nissle 1917, *Enterococcus faecium* SF68, and *Saccharomyces boulardii*. Probiotics are different from prebiotics, which are non-viable food components that confer health benefits on the host through modulation of the microbiota. They are a group of diverse carbohydrate ingredients, most commonly in the form of non-digestible oligosaccharides. Synbiotics are a combination of prebiotics and probiotics [9].

Although some studies show promise for the use of probiotics in treating food allergy, the evidence is still conflicting and inconclusive [10]. Most systematic reviews conducted on probiotics and food allergy focused on the role of probiotics in preventing food allergy [11–15]. In 2015, the World Allergy Organization with McMaster University created a guideline for allergic disease prevention using probiotics. Probiotic use among infants at high risk of allergic disease was conditionally recommended due to its net benefit of preventing eczema; however this had very low quality of evidence [16].

A systematic review conducted in 2013 found 11 studies on the use of probiotics for treating atopic disease—1 systematic review, 8 randomized controlled trials, and 2 non-randomized trials. These 11 studies included 6 studies on food allergy, 3 studies on atopic dermatitis, 1 study on birch pollen allergy, and 1 study on atopic disease in general. It did not conduct a meta-analysis of the results [17]. This systematic review aims to synthesize the available evidence on the use of probiotics as treatment of food allergy among pediatric patients.

## Research question

Among pediatric patients with food allergy, how effective is probiotic administration with standard therapy compared to standard therapy alone in the relief of allergic symptoms?

## Objectives

### General objective

To determine the effectiveness of probiotic administration in treating food allergies among pediatric patients.

### Specific objectives

1. To determine the effect of probiotic administration on the relief of allergic symptoms among pediatric patients with food allergy
2. To determine the effect of probiotic administration on the inducement of tolerance among pediatric patients with food allergy
3. To determine adverse events associated with probiotic administration among pediatric patients with food allergy
4. To perform subgroup analysis on the effectiveness of probiotics among the different probiotic strains, types of food allergy, and time periods in measuring the outcome.

## Methods

### Selection criteria

#### Inclusion criteria

##### Types of studies

All randomized controlled trials (RCT) available from inception until February 19, 2018 on probiotics as treatment for food allergy among pediatric patients were included in this systematic review. We included studies published in any language.

##### Types of participants

Studies involving pediatric patients with any type of food allergy (cow’s milk allergy, egg allergy, peanut allergy, fish allergy, shellfish allergy, wheat allergy, soy allergy), were included. The diagnosis of food allergy was confirmed through double-blind placebo-controlled food challenges. Patients were said to have suspected food allergy if purely clinical diagnosis was done. Studies involving participants with confirmed and suspected food allergy were included in this review.

##### Types of interventions

Studies involving oral administration of probiotics, regardless of strain and dose, were included. The control is placebo with standard management of food allergy.

##### Types of outcome measures

The primary outcome of interest is the relief of allergic symptoms. This is commonly measured through a scoring system called the SCORAD (Scoring Atopic Dermatitis) index, which is a clinical tool to assess the extent and severity of eczema. The SCORAD index takes into consideration erythema, edema/papulation, oozing/crusting, excoriations, lichenification, dryness, pruritus, and sleep interference. The SCORAD index score ranges from 0 to 103 [18]. Relief of allergic symptoms can also be reported in a binary manner, as presence or absence of allergic manifestations during a specified time period.

The secondary outcome of interest is the inducement of tolerance. Tolerance is defined as the state of healthy unresponsiveness to food allergens. Patients with food allergy are said to have acquired tolerance to the food allergen if there is absence of allergic symptoms after consumption of the food allergen or upon oral food challenge [1, 6]. This is measured in a binary manner, as presence or absence of tolerance during a specified time period. Another outcome of interest is the development of adverse events associated with probiotic administration, measured as presence or absence of adverse outcomes.

#### Exclusion criteria

Studies that reported only surrogate outcomes such as immunoglobulin determination, cytokine levels, lymphocyte counts, or other biochemical markers were excluded from the analysis. Studies involving adult patients and patients with atopic diseases other than food allergy were excluded in the review.

### Search strategy

A systematic search of electronic medical literature databases including Cochrane Library, MEDLINE, TRIP Database, and Herdin was conducted. The principal free text search terms used were: "probiotics" and "food allergy" or "food hypersensitivity" or "food anaphylaxis". The Medical Subject Heading terms used were: "Probiotics" and "Food Hypersensitivity". The Highly Sensitive Search Strategy for identifying randomized trials in Medline from the Cochrane Handbook was done [19]. Comprehensive manual search of the reference lists of the retrieved articles was also conducted.

Unpublished articles were explored by writing to experts, corresponding with pharmaceutical industries, and surveying conference proceedings and books of abstracts. Registries of clinical trials were likewise searched, including the World Health Organization Network of Collaborating Clinical Trial Registers and the U.S. National Institutes of Health website, clinicaltrials.gov.

### Data collection and analysis

#### Study selection

Two independent authors reviewed the studies collected from the electronic and manual searches. Initial screening through evaluation of the titles and abstracts was done. Studies which matched the pre-specified selection criteria were included in the second screening, where the full-text articles were retrieved and appraised.

Disagreements from the screening process were discussed until resolved. If consensus was not reached, an independent third party reviewer was consulted. A sample of the screening form used is shown in Additional file 1: Appendix S1.

#### Data extraction and management

The following data were extracted from each study by two independent reviewers: 1.) author, 2.) year of publication, 3.) setting, 4.) study population size, 5.) type of food allergy, 6.) probiotic strain, 7.) control used, and 8.) outcome evaluated. In case of disagreement, the reviewers consulted each other to arrive at a consensus. If consensus was not reached, an independent third party reviewer was consulted. A sample of the data abstraction form used is shown in Additional file 1: Appendix S2.

#### Assessment of risk of bias in included studies

All included studies were independently appraised using the Cochrane risk of bias tool [18] by two review authors. The following parameters were evaluated: random sequence generation, allocation concealment, blinding of participants and personnel, blinding of outcome assessment, incomplete outcome data, and selective reporting. In case of disagreement, the reviewers consulted each other to arrive at a consensus. If consensus was not reached, an independent third party reviewer was consulted.

All studies were included in the systematic review regardless of level of appraisal. Sensitivity analysis excluding studies with high risk of bias in the domains of randomization, blinding of participants, personnel or outcome assessment, or attrition, was done to evaluate the impact of these studies on the over-all results.

A risk of bias summary and graph was generated using RevMan 5.3.

#### Measures of treatment effect

We evaluated the pooled effect for relief of allergic symptoms through the inverse variance method using the mean difference and standard deviation (if the included studies used the same scale) or standardized mean difference (if studies used different scales). Binary outcomes (inducement of tolerance and adverse outcomes) were combined across studies using Mantel-Haenszel method and expressed as risk ratios (RR) with 95% confidence interval (CI).

#### Unit of analysis issues

The main unit of analysis is the study participants. Some studies evaluated tolerance on multiple time periods for the same group of patients. In this situation, the authors considered the number of patients who failed to acquire tolerance for each time period as the events. The number of participants in the group at the start of the study was considered the total number of participants for each time period. One study evaluated the SCORAD index on multiple time periods. The SCORAD index scores in the different time periods were all compared to baseline SCORAD index scores.

#### Dealing with missing data

Presence of dropouts was determined in each study. Greater than 20% dropout was considered significant. We conducted worst-case scenario sensitivity analysis to determine whether the effect of treatment would be reversed. Studies whose effects reversed in the worst-case scenario were considered to have high risk of attrition bias. In the pooling of results, analysis was based only on the available data.

#### Assessment of heterogeneity

Heterogeneity was quantified using chi-square tests and the inconsistency statistic (I^2^). Studies with I^2^ > 50% and *p* < 0.1 were considered to have significant heterogeneity. If there was no significant heterogeneity, analysis was done using fixed-effects model. Random-effects model was used if there was significant heterogeneity. Subgroup analysis was done to explore the source of heterogeneity.

#### Data synthesis

Data analysis and synthesis were done using RevMan 5.3 software. Subgroup analysis was done for different probiotic strains, types of food allergies, and time periods in measuring the outcome. Post-hoc subgroup analysis was also conducted to evaluate the effect of probiotics on confirmed CMA and suspected CMA patients.

Exclusion sensitivity analysis was done to evaluate the stability of the primary outcome of the study.

## Results

### Description of studies

#### Results of the search

**Fig. 1:**
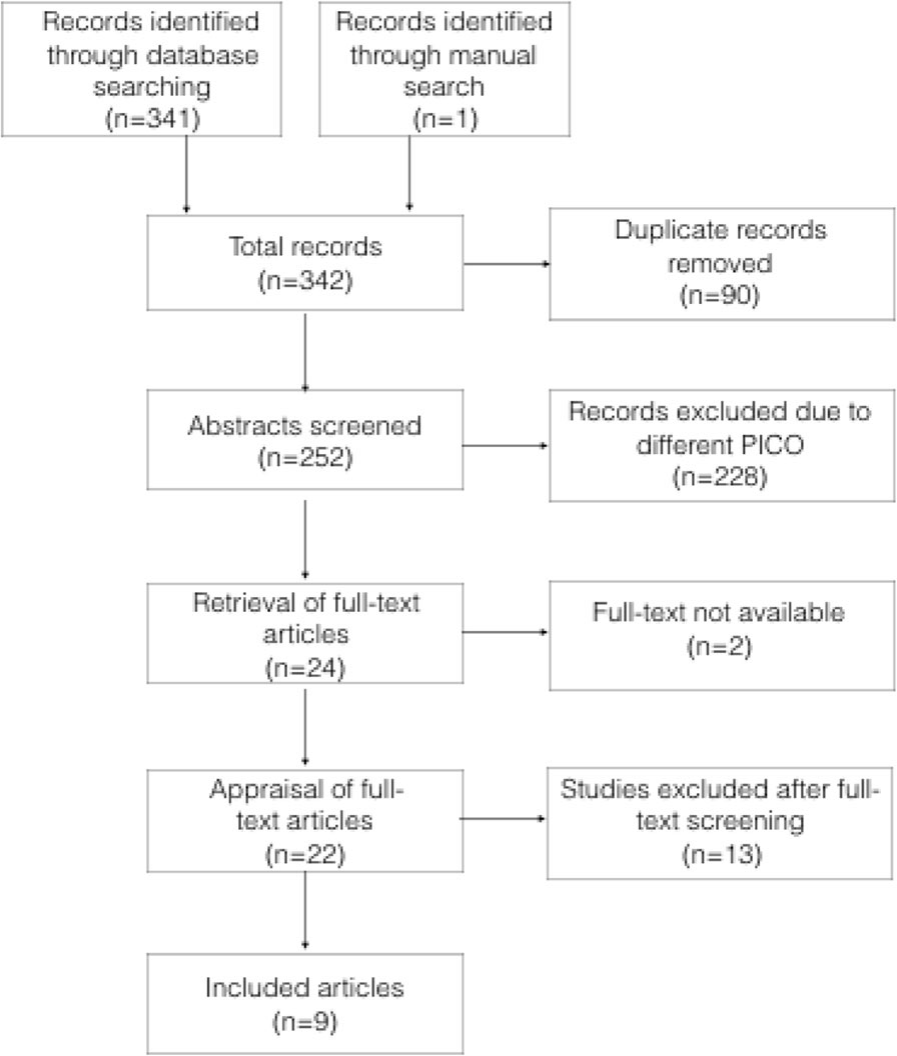
Search flow diagram

A total of 342 articles were identified in the search. There were 90 duplicate records. After screening of abstracts, 228 records were excluded because they did not match the selection criteria or the population, intervention, comparison, and outcome (PICO) specified for this systematic review. The full-text articles of the remaining 24 studies were retrieved and assessed for eligibility. The full-text of two Russian studies were not retrieved despite thorough search of the article and the authors’ contact details. Out of the 22 studies included in the second screening, nine studies were included in this systematic review and meta-analysis. The search flow diagram is shown in Fig. 1.

The detailed database search is shown in Additional file 1: Appendix S3. Three pharmaceutical companies and one expert responded that they are not aware of unpublished trials on this topic.

#### Included studies

Nine studies [20–28] with a total of 895 pediatric patients aged 1 month to < 2 years of age from both sexes were included. All studies were randomized, placebo-controlled trials on the use of probiotics for treating food allergy. Eight studies were in English, one study [23] was in Polish. The Polish article was translated to English using Google Translate.

All studies involved participants with CMA. Three studies [23–25] involved patients with confirmed diagnosis of CMA while the remaining six studies involved patients with suspected CMA.

Five studies [20–22, 26, 27] evaluated *Lactobacillus rhamnosus* GG (LGG). Two studies [24, 25] used a probiotic mixture containing *L. casei* CRL431 and *Bifidobacterium lactis* Bb-12. One study [23] used a probiotic mixture containing *L. casei* LOCK 0900, *L. casei* LOCK 0908 and *L. paracasei* LOCK 0919. One study [28] was a multi-arm trial, with one arm given LGG, another given mixed probiotic containing LGG, *L. rhamnosus* LC705, *Bifidobacterium breve* Bbi99, and *Propionibacterium freudenreichii* ssp. *shermanii* JS, and the control group on placebo.

Five studies reported the SCORAD index values [23, 24, 26–28]; four studies reported tolerance [21–23, 25]. One study [23] reported both SCORAD index and tolerance. Two studies reported persistence of allergic symptoms [20, 22].

The summary of the characteristics of the included studies is presented in Additional file 1: Appendix S4.

#### Excluded studies

Out of the 22 full-text articles, 13 were excluded since they did not meet the inclusion criteria [29–41]. Six articles were excluded because they reported only surrogate markers (fecal butyrate concentration, skin prick test, urinary organic acid concentration, levels of IgE, IgA, natural killer cells). One study was excluded because the outcome was hypoallergenicity of milk formula with probiotics, not the effectiveness in treating food allergy. Three articles did not have children with food allergy as their study population. Two articles evaluated synbiotics, while one article had a co-intervention of immunotherapy. Two studies administered probiotics to their control group. The summary of the characteristics of the excluded studies is shown in Additional file 1: Appendix S5.

### Risk of Bias in included studies

**Fig. 2:**
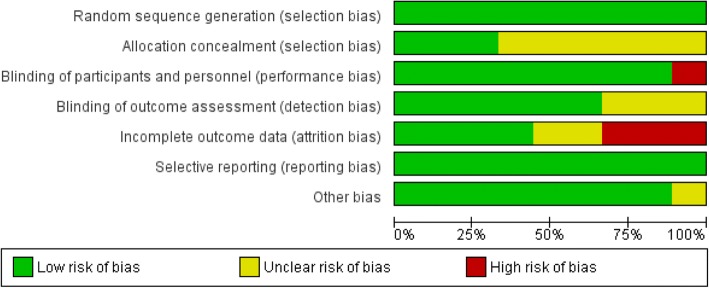
Risk of bias graph

**Fig. 3:**
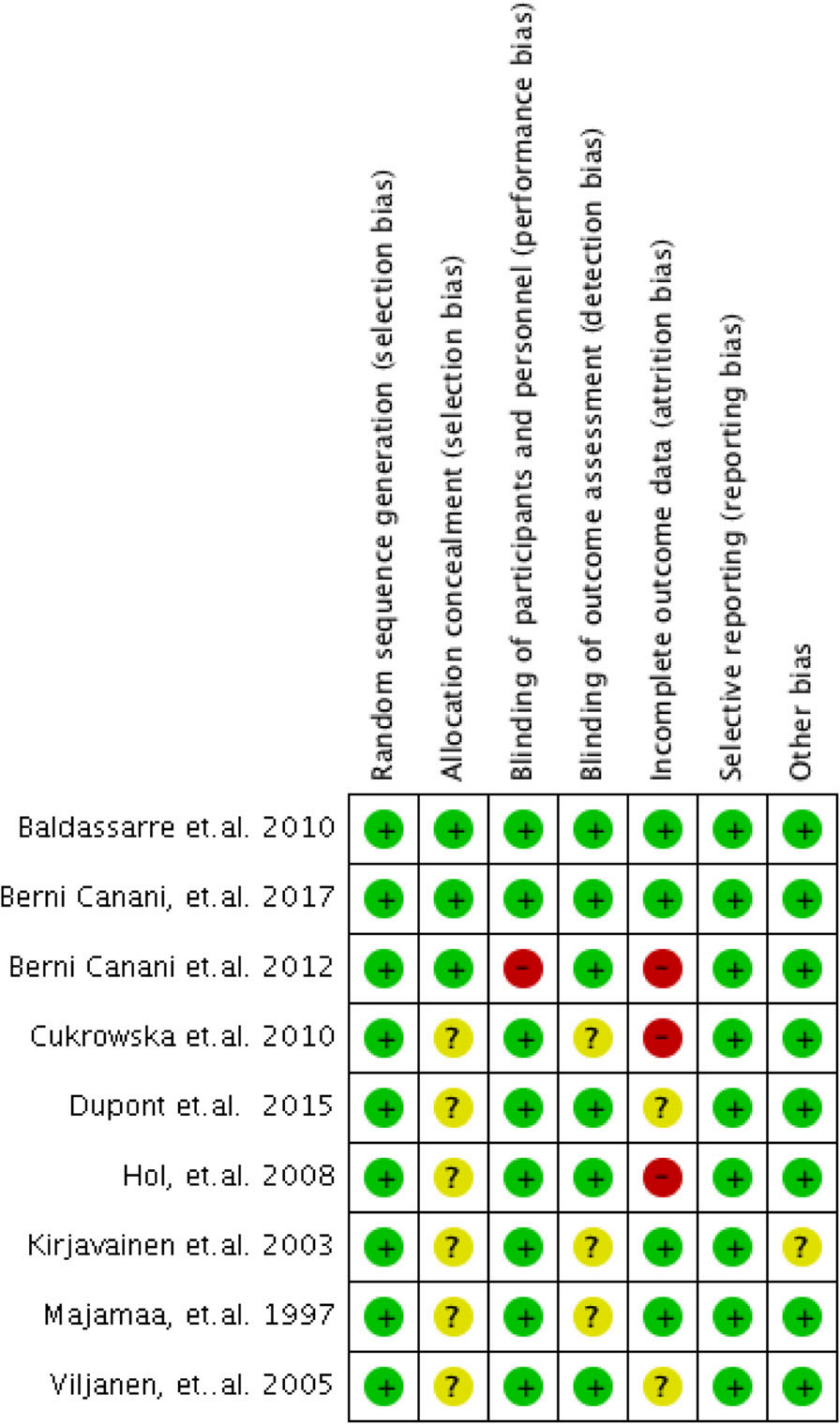
Risk of bias summary

The risk of bias graph and summary are presented in Figs. 2 and 3. All trials utilized randomization. Most trials did not provide adequate information on allocation concealment. The authors were contacted via email; however, only one author verified how allocation concealment was done. Eight studies reported blinding of patients and health care professionals by using placebos identical in appearance to the intervention. One study [21] was an open study and did not perform blinding of participants and personnel; thus, this study was considered to have high risk of bias. Blinding of outcome assessors was not clearly reported in some studies [23, 26, 27]. These authors were contacted, but no response was received at the time of writing of this paper. Three studies [21, 23, 25] had high dropout rates where worst-case scenario sensitivity analysis showed reversal of conclusions; hence, these were assessed to have high risk of attrition bias. Sensitivity analysis could not be done for two studies [24, 28] due to inadequate data, but the drop-out rate was less than 20%. One study [26] was terminated early and blinding was broken due to safety issues. It was unclear in the report if the one who did data analysis remained blinded or not. The authors were contacted, but no response was received at the time of writing this paper.

Visualization of the funnel plot to assess publication bias could not be done since there were less than 10 studies included in this review.

An over-all assessment of the evidence was performed using GRADE, as shown in Table 1.

**Table 1:** Evidence Profile

Certainty assessment							№ of patients		Effect		Certainty	Importance
No. of studies	Study design	Risk of bias	Inconsistency	Indirectness	Imprecision	Other considerations	Probiotics	Placebo	Relative (95% CI)	Absolute(95% CI)		
SCORAD Index (assessed with: mean difference)												
2	randomized trials	not serious	not serious	not serious	serious ^a^	none	209	205	–	MD 1.3 lower (3.88 lower to 1.28 higher)	⨁⨁⨁◯ MODERATE	CRITICAL
No tolerance (assessed with: relative risk)												
4	randomized trials	serious ^b^	not serious	not serious	serious ^a^	none	40/153 (26.1%)	73/161 (45.3%)	RR 0.70 (0.39 to 1.26)	136 fewer per 1000 (from 118 more to 277 fewer)	⨁⨁◯◯ LOW	CRITICAL

*CI* Confidence interval, *RR* Risk ratio, *MD* Mean difference

^a^Serious imprecision due to wide confidence intervals straddling the no-effect line

^b^High risk of attrition bias in 3 out of 4 studies

### Effects of interventions

#### Effect of probiotic administration on relief of allergic symptoms

Two studies reported the mean difference and standard deviation of the SCORAD index [24, 28]. The study of Viljanen [28] was a multi-arm trial evaluating two types of probiotics with placebo, so there were two data entries from this study in the meta-analysis. Viljanen measured the outcome at 2 months, while Dupont measured the outcome at 6 months.

Two studies [23, 26] reported the mean difference of the SCORAD index, but the standard deviation was not given and could not be derived given the available data. The study of Cukrowska [23] reported a greater reduction in the mean SCORAD index of the probiotics group compared to the placebo group at 3 months (37.4 point reduction for probiotics, 10 point reduction for placebo), 8 months (30.9 points versus 25.3 points), and 24 months (40.7 points versus 34.72 points), thus favoring the use of probiotics in relieving eczema. The study of Kirjavainen [26] also reported greater reduction in mean SCORAD index of the probiotics group compared to the placebo group at 2 months (14 point reduction versus 5 point reduction).

The study by Majamaa [27] reported the median and interquartile range of the SCORAD index. At 1 month, the probiotics group had an 11 point decrease in their median SCORAD index compared to the 2 point decrease in the placebo group. Requests were sent to the authors for additional data so that their study results may be pooled; however, no response was received at the time of writing this paper.

**Fig. 4:**
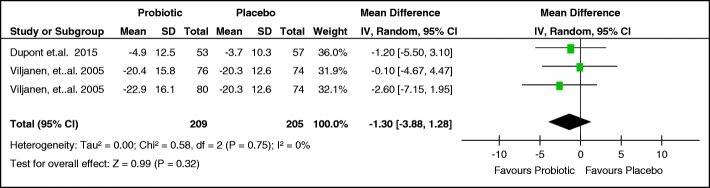
Effect of probiotics compared to placebo on the mean SCORAD index

Only data from the Dupont and Viljanen studies [24, 28] were pooled using RevMan 5.3. The pooled mean difference is − 1.3 (95% CI -3.88, 1.28) with an over-all effect z-score of 0.99 (*p*-value 0.32). Although the mean difference favors probiotic use, the CI is wide and includes the no effect line; hence, the results are not precise. There was no significant heterogeneity with Chi^2^ = 0.58, *p* = 0.75 and I^2^ = 0% (Fig. 4).

The study by Baldassarre [20] reported persistence of fecal occult blood in 64.3% of infants in the placebo group, compared to 0% in the probiotic group (*p* = 0.027). The relative risk was 0.06 (95% CI 0.004, 0.94). The 2017 study by Berni Canani [22] reported lower frequency of allergic manifestations, including eczema, urticaria, asthma, and rhinoconjunctivitis, in the probiotics group compared to the control group. The relative risk was 0.51 (95% CI 0.33, 0.77) with a *p*-value of 0.001. Since these two studies reported the effect of probiotics on different allergic manifestations, the data was not pooled.

#### Effect of probiotic administration on tolerance

**Fig. 5:**
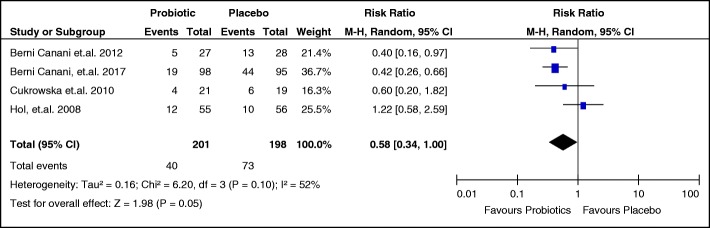
Effect of probiotics compared to placebo on failure to acquire tolerance

The secondary outcome of interest, tolerance, was reported in four studies [21–23, 25]. The review authors considered the number of participants who failed to acquire tolerance as the event of interest. The pooled results of these studies revealed an RR of 0.58 (95% CI 0.34, 1.00) and an over-all effect z-score of 1.98 (*p* = 0.05). While the point estimate favors the use of probiotics in inducing tolerance among children with food allergy, this result is imprecise since the CI includes the no effect line. Furthermore, the studies exhibit significant heterogeneity with I^2^ of 52%. The forest plot showing the summary of the results is shown in Fig. 5.

Sensitivity analysis was conducted to determine the treatment effect when studies with high risk of bias were excluded. Since three studies [21, 23, 25] have high risk of attrition bias, and the 2012 study by Berni Canani [21] has high risk of performance bias, only one study was left [22]. This study showed effectiveness of probiotics compared to placebo in inducing tolerance among children with food allergy, with a precise CI (RR 0.42, 95% CI 0.26, 0.66).

**Fig. 6:**
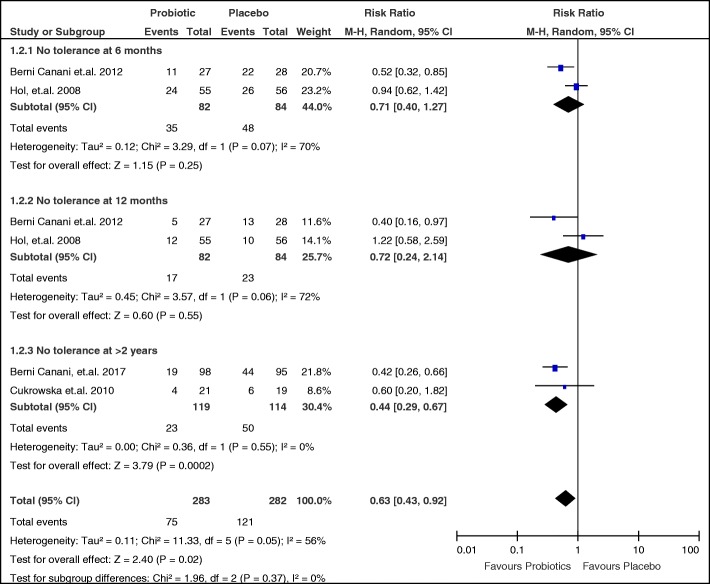
Subgroup analysis on failure to acquire tolerance per time period

Subgroup analysis based on the time period of measurement of tolerance was done (Fig. 6). Two studies [21, 25] were pooled at 6 months and 12 months while two other studies were pooled at ≥2 years with Cukrowska [23] reporting tolerance at 2 years and Berni Canani [22] at 3 years. At 6 months, the RR is 0.71 (95% CI 0.40 to 1.27), with an over-all effect of z = 1.15 and *p*-value = 0.25. Although the point estimate favors probiotic use, the CI is not precise. At 12 months, the RR is 0.72 (95% CI 0.24 to 2.14) with an over-all effect z = 0.60 and *p*-value of 0.55. The point estimate still favors probiotics, but again the CI remained imprecise. Moreover, heterogeneity remained significant at I^2^ of 70% and 72% for 6 months and 12 months respectively. For tolerance ≥2 years, the RR is 0.44 (95% CI 0.29 to 0.67) with an over-all effect z = 3.79 and *p*-value of 0.0002. The point estimate favors probiotic use with a precise CI and no significant heterogeneity with I^2^ of 0%.

**Fig. 7:**
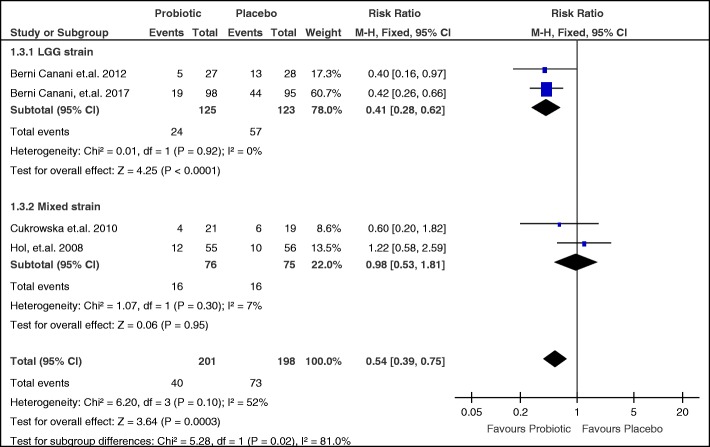
Subgroup analysis of probiotic strains on failure to acquire tolerance

Subgroup analysis on type of probiotic strain was also done (Fig. 7). Two studies that evaluated LGG [21, 22] were combined, with pooled RR of 0.41 (95% CI 0.28 to 0.62), over-all effect z = 4.24 and *p*-value < 0.001. The point estimate favors probiotics, with a precise CI. There was no heterogeneity, with I^2^ of 0% and p-value of 0.92. The two other studies [23, 25] used mixed probiotic strains. The pooled RR is 0.98 (95% CI 0.53 to 1.81) with an over-all effect z = 0.06 and p-value of 0.95. The point estimate slightly favors probiotics, but the CI is not precise. There was no significant heterogeneity with I^2^ of 7% and p-value of 0.30.

**Fig. 8:**
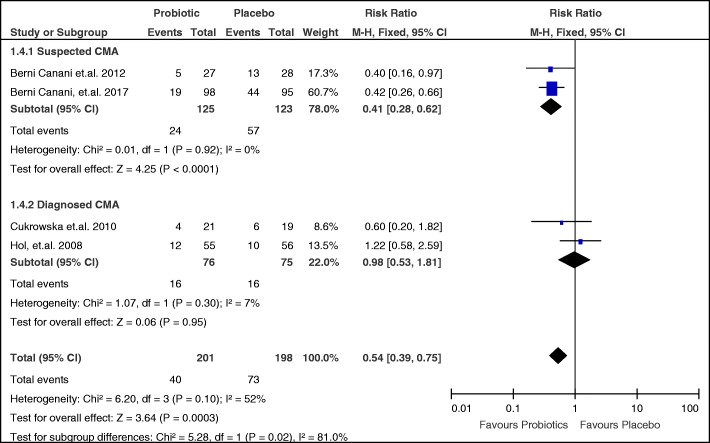
Subgroup analysis on failure of acquisition of tolerance among diagnosed and suspected CMA patients

Post-hoc subgroup analysis on the effect of probiotics for suspected CMA and diagnosed CMA patients was also done (Fig. 8). The same 2 studies [21, 22] had suspected CMA as their study population and the same two studies [23, 25] had diagnosed CMA patients as their study population hence, the same pooled RR, CI, and heterogeneity values as above will be obtained.

#### Adverse effects of probiotics administration

Five studies [21–23, 25, 26] reviewed adverse effects of probiotics. Only one study [26] reported presence of adverse effects, but this was among patients given heat-inactivated LGG where 38% of the study participants (5 out of 13 infants) developed prolonged diarrhea. This caused the study to be prematurely terminated and blinding to be broken. No adverse events were reported in the treatment group given viable LGG. Heat inactivation may have possibly caused modification of immunostimulatory properties of the LGG due to denaturation of the surface peptides. The other four studies reported that probiotics was tolerated well by the study participants.

## Discussion

### Summary of Main results

The main results of this systematic review are shown in the evidence profile and summary of findings table generated using Gradepro GDT (Tables 1 and 2).

**Table 2:** Summary of Findings

Outcomes	Anticipated absolute effects^*^ (95% CI)		Relative effect (95% CI)	№ of participants (studies)	Certainty of the evidence (GRADE)	Comments
	Risk with placebo	Risk with Probiotics				
SCORAD index assessed with: mean difference	The mean new outcome was 0	The mean new outcome in the intervention group was 1.3 lower (3.88 lower to 1.28 higher)	–	414 (2 RCTs)	⨁⨁⨁◯ MODERATE ^a^	
No tolerance (Failure to acquire tolerance) assessed with: relative risk	453 per 1000	317 per 1000 (177 to 571)	RR 0.70 (0.39 to 1.26)	314 (4 RCTs)	⨁⨁◯◯ LOW ^a,b^	

*CI* Confidence interval, *RR* Risk ratio, *MD* Mean difference

^a^Serious imprecision due to wide confidence intervals straddling the no-effect line

^b^High risk of attrition bias in 3 out of 4 studies

This meta-analysis summarized the available evidence on use of probiotics for treating children with food allergy. Only studies on CMA were analyzed since no studies were found on probiotics as treatment for other types of food allergy among children.

In general, the pooled risk ratio from the studies favors the use of probiotics in CMA in reducing SCORAD scores and inducing tolerance, but the wide CI indicates that probiotics may in fact have no difference from placebo and precludes definite conclusions to be made. These findings are moreover tempered by significant heterogeneity. Although probiotics have been shown to have immunomodulating effects on humans [8, 42, 43], pooled evidence from available RCTs do not definitively demonstrate this effect. Good quality studies with larger sample size are needed to narrow the confidence interval.

Although there was general trend of improvement in the SCORAD scores among the five included studies, the lack of available data limited pooling of data from three studies. The study of Viljanen [28] had two subsets of probiotics—LGG alone and a mixture of probiotics. It was hypothesized that the mixed probiotics will reinforce the beneficial effects of LGG alone. However, there was smaller mean reduction of SCORAD scores in the mixed probiotics group compared to the LGG group, pointing to the superior effect of LGG alone compared to the mixed strains. This could possibly be due to inhibitory interactions between probiotic strains, which have been observed in other studies. Inhibition may occur through production of antimicrobial substances by the probiotic strain, or competition for the same nutrients by the probiotic strains, which would reduce the efficacy of mixed probiotic strains [44]. Unfortunately, this observation is constrained by the wide CI for both the LGG and mixed group.

Based on a pre-planned subgroup analysis for probiotic strains, pooled studies with moderate quality show that the LGG strain is effective in inducing tolerance among infants with suspected CMA. This finding illustrates the variation of treatment effect depending on the probiotic strain or mixture of strains used.

Another pre-planned subgroup analysis noted significant benefit of probiotic administration in inducement of tolerance at 2 years or more. This finding suggests a possible duration-dependent effect of probiotic use, with probiotics significantly increasing inducement of tolerance among children with food allergy in the long term.

### Overall completeness and applicability of evidence

The objectives of this meta-analysis were sufficiently addressed at the end of the review. All included studies involved patients with CMA whose baseline characteristics were not significantly different. Majority of the studies were done in European countries (Finland, Netherlands, Poland, Italy), while one study was done in the United States. All studies used probiotics for treatment of CMA, and the outcome measures reported were improvement of allergic symptoms, tolerance or both. The results of this review can be applied to similar populations as those included in the studies. Extrapolating the results of this meta-analysis to other countries may be difficult, given the wide variation of probiotic strains available commercially in each country or region. As evidenced by the significant change in the effect size when subgroup analysis per type of probiotic strain was done, the utility of probiotic to treat food allergy is largely affected by the type of probiotic used.

### Quality of evidence

The review presented nine randomized placebo-controlled studies which were critically appraised and assessed to have over-all moderate quality of evidence, taking into consideration selection, performance, detection, attrition and reporting biases. Thus, the results of this systematic review are valid and applicable.

### Potential biases in the review process

Selection bias was controlled by clearly establishing and following the inclusion and exclusion criteria during the search of articles. Only randomized controlled trials were included in this review. Two authors conducted independent systematic search and screening of the articles. Disagreements were resolved through discussion. Publication bias was controlled by searching for unpublished articles through writing to experts, correspondence with pharmaceutical industries, and surveying conference proceedings and books of abstracts. Furthermore, the authors of the included studies were contacted to verify and complete the data needed for the review.

### Agreements and disagreements with other studies or reviews

The results of this systematic review is somewhat in agreement with the results of the previous systematic review conducted in 2013 [17], which stated that probiotics have not been proven helpful in treating food allergy. The conclusion of the 2013 review was based on studies with contrasting results showing benefit and no benefit of probiotics.

Subgroup analysis in this systematic review, however, showed significant benefit of LGG administration in inducing tolerance, as well as a duration-dependent effect with significant benefit in inducing tolerance noted after at least 2 years.

## Conclusions

### Implications for practice

This systematic review and meta-analysis show moderate certainty that the use of probiotics can relieve symptoms of children with cow’s milk allergy. The reduction in certainty is due to imprecise results. Moreover, there is low certainty that probiotics can induce tolerance among children with cow’s milk allergy, due to problems with imprecision and attrition bias. In the subgroup analysis, *Lactobacillus rhamnosus* GG administration likely results in inducing tolerance among infants with suspected cow’s milk allergy. Another subgroup analysis also show duration-dependent effect associated with giving probiotics, where it can induce tolerance after at least 2 years.

### Implications for research

It is recommended that more research should be done on other common types of food allergy such as peanut, egg, wheat and shellfish. More studies with larger sample size are needed to clearly show beneficial effects of probiotics in treating food allergy. Studies that compare the effectiveness of different probiotic strains for treatment of food allergies, especially those that are available commercially, should be done. A standard way of reporting observations and results is also recommended to enable pooling of more data for future reviews.

### Differences between protocol and review

The review contains post-hoc subgroup analysis on suspected CMA and diagnosed CMA infants. During data extraction, the authors noted that some studies involved participants diagnosed with CMA through the double-blind placebo-controlled food challenge, which is the gold standard. Some studies involved participants suspected to have CMA through clinical diagnosis alone. Subgroup analysis was done to evaluate the possible effect of this difference in population. The rest of the review is in accordance with the protocol created by the authors.

## Additional file

Additional file 1: **Appendix S1.** Sample Screening Form. **Appendix S2.** Sample Data Abstraction Form. **Appendix S3.** Database Search. **Appendix S4.** Characteristics of Included Studies. **Appendix S5.** Characteristics of Excluded Studies. (DOCX 38 kb)

## Abbreviations

CI: Confidence interval
CMA: Cow’s milk allergy
FAO: Food and Agriculture Organization
I^2^: Inconsistency statistic
LGG: *Lactobacillus rhamnosus* GG
PICO: Population, intervention, comparison, outcome
RCT: Randomized controlled trial
RR: Risk ratio
SCORAD: Scoring atopic dermatitis
WHO: World Health Organization
