# Supplementary material for: Probiotics as treatment for food allergies among pediatric patients: a meta-analysis
Source: World Allergy Organ J. 2018 Nov 6;11(1):25. doi: 10.1186/s40413-018-0204-5 (PMC6218986; doi:10.1186/s40413-018-0204-5)
Supplement: Supplementary file 1 — Appendix S1. Sample Screening Form. Appendix S2. Sample Data Abstraction Form. Appendix S3. Database Search. Appendix S4. Characteristics of Included Studies. Appendix S5. Characteristics of Excluded Studies. (DOCX 38 kb) [file 40413_2018_204_MOESM1_ESM.docx]

**Additional file 1**

- 1. **Appendix S1: Sample Screening Form**

**Screening Eligibility Form**

Title of Review:

Reference Citation:

| Selection Criteria | | Yes | No | Unclear | Comments |
| --- | --- | --- | --- | --- | --- |
| Is the study a randomized controlled trial? | |  |  |  |  |
| Is the study population on children diagnosed with food allergy? | |  |  |  |  |
| Is the treatment intervention probiotics? | |  |  |  |  |
| Is the control group any of the following: placebo, standard management of allergy (avoidance of allergen, symptomatic treatment) | |  |  |  |  |
| Are the outcome measures any of the following:  1. Primary treatment outcomes  Persistence of allergic symptoms  2. Secondary treatment outcomes:  Failure to acquire tolerance | |  |  |  |  |
| Decision: | INCLUDE  EXCLUDE  Reason for exclusion ________________________________________  Retrieve full text | | | | |

Assessed by: _____________________________________

Date: _____________________________________________

- 1. **Appendix S2: Sample Data Abstraction Form**

**Data Extraction Template**

| Trial ID | Extractor | Year of publication |
| --- | --- | --- |
| Title | | |
| Authors | | |

# Trial Methods

| Method of random sequence  generation | Describe: Adequate / unclear / inadequate |
| --- | --- |
| Method of allocation  concealment | Describe: Adequate / unclear / inadequate |
| Blinding | Participant: yes / no / unclear / not feasible  Clinician: yes / no / unclear / not feasible  Outcome assessor : yes / no / unclear / not feasible |
| Loss of participants to follow up | Describe: 5-9.9% > 10-19.9% > 20% |
| Incomplete outcome data / intention to treat analysis | Describe: Used / unclear / not used |
| Selective reporting: | Describe Yes/no/unclear |
| Other bias | Describe Yes/no/unclear |

# Participants

**Inclusion criteria:**

**Number of participants:**

**Age range of participants:**

**Exclusion criteria:**

# Intervention

**Experiment group:**

**Control group:**

# Additional information requested

# Notes

**Outcomes**

|  | Outcome Measures (Dichotomous) | Total participants = | | | |
| --- | --- | --- | --- | --- | --- |
|  |  | Intervention group  n = | | Control group  n = | |
|  |  | events | total | events | total |
|  | Primary: |  |  |  |  |
| 1 |  |  |  |  |  |
|  | Secondary: |  |  |  |  |
| 2 |  |  |  |  |  |
| 3 |  |  |  |  |  |

|  | Outcome Measures (Continuous) | Total participants = | | | | | |
| --- | --- | --- | --- | --- | --- | --- | --- |
|  |  | Intervention group  n = | | | Control group  n = | | |
|  |  | total | mean | SD | total | mean | SD |
|  | Primary: |  |  |  |  |  |  |
| 1 |  |  |  |  |  |  |  |
|  | Secondary: |  |  |  |  |  |  |
| 2 |  |  |  |  |  |  |  |
| 3 |  |  |  |  |  |  |  |

- 1. **Appendix S3: Database Search**

1. **MEDLINE (Feb 20, 2018)**

A total of 128 articles were found. 5 were done in adults, 34 were not related to food allergy (IBS, atopic dermatitis, eczema, etc.), 37 were prevention studies. Seven articles were not about probiotics (prebiotics, amino acid formula, oligosaccharides), 27 articles were not RCTs (review, editorial, etc.). A total of 18 articles were for retrieval, but 2 articles could not be retrieved. Sixteen full-text articles were retrieved for review.

1. **Cochrane Library (February 20, 2018)**

A total of 134 studies were found. Three studies were done in adults, 29 were not related to food allergy (sepsis, abdominal pain, diarrhea, cancer, urinary tract infection, etc.), 10 were prevention studies. Ten articles were not about probiotics (immunotherapy, formula milk, vaccines, prebiotics, etc.), 1 article was not an RCT (review), 1 did not use standard of care as control (soy milk). There were 76 duplicate entries. Four articles were retrieved for review.

1. **Trip Database (February 20, 2018)**

The search terms used in Trip Database were ‘probiotic’ and ‘food allergy’. A total of 35 studies were found. Two studies were done in adults while 1 was done with mice as subjects. One was not related to food allergy (birch pollen allergies). Seven were not RCTs (review, cost-effectiveness study, editorial), 9 were regarding prevention studies and not treatment. Two studies were study protocols which were withdrawn/terminated. There were 12 duplicate entries from Medline and Cochrane. One study was retrieved for review.

1. **Herdin (February 21, 2018)**

The search terms used in Herdin were ‘probiotic’ and ‘food allergy’ and ‘randomized controlled trial’. A total of 44 studies were found. Three studies were done in adults while 8 were done in animals. Seven were not related to food allergy (obesity, diabetes, liver cirrhosis, malnutrition, etc.), 23 were not on probiotics (dibencozide, seaweed, garlic, pineapple juice, etc.), 1 was a prevention study. There were 2 duplicate entries within Herdin. No studies were retrieved for review.

- 1. **Appendix S4: Characteristics of Included Studies**

| **STUDY** | **Country** | **Type of Study** | **No. of participants** | **Population** | **Type of food allergy** | **Type of Probiotic** | **Outcome measure** |
| --- | --- | --- | --- | --- | --- | --- | --- |
| Baldassarre et al. 2010 [20] | USA | RCT | 26 | Infants 0- 12 months old | Suspected CMA | LGG | Positive occult blood stool |
| Berni Canani et al. 2012 [21] | Italy | RCT | 55 | Infants 1-12 months old | Suspected CMA | LGG | Tolerance |
| Berni Canani et al.  2017 [22] | Italy | RCT | 220 | Infants 1 to 12 months old | Suspected CMA | LGG | Persistence of symptoms  Tolerance |
| Cukrowska et al. 2010 [23] | Poland | RCT | 60 | Children less than 2 years | Confirmed CMA | *Lactobacillus casei* LOCK 0900,  *Lactobacillus casei* LOCK 0908,  *Lactobacillus paracasei* LOCK 0919 | SCORAD index  Tolerance |
| Dupont et al. 2015 [24] | Netherlands | RCT | 119 | Infants less than 6 months | Confirmed CMA | *Lactobacillus casei* CRL431 and *Bifidobacterium lactis* Bb-12 | SCORAD index |
| Hol et al. 2008 [25] | Netherlands | RCT | 119 | Infants less than 6 months | Confirmed CMA | *Lactobacillus casei* CRL431 and *Bifidobacterium lactis* Bb-12 | Tolerance |
| Kirjavainen et al. 2003 [26] | Finland | RCT | 35 | Infants 3.5-6.8 months old | Suspected CMA | LGG (Viable LGG vs. heat-inactivated LGG) | SCORAD index |
| Majamaa et al. 1997 [27] | Finland | RCT | 31 | Children 2.5 to 15.7 months | Suspected CMA | LGG | SCORAD  index |
| Viljanen et al. 2005 [28] | Finland | RCT | 230 | Children 1.4 to11.9 months old | Suspected CMA | 1. LGG  2. LGG + *Lactobacillus rhamnosus* LC 705 +  *Bifidobacterium breve* + *Propionibacterium freudenreichii* ssp. *shermanii* | SCORAD index |

- 1. **Appendix S5: Characteristics of Excluded Studies**

| **STUDY** | **Reason for exclusion** |
| --- | --- |
| Ahanchian et al. 2013 [29] | Intervention included synbiotics, not probiotics |
| Burks et al. 2015 [30] | Intervention included synbiotics, not probiotics |
| Berni Canani et al. 2016 [31] | Outcome measured was only a surrogate marker (fecal butyrate concentration) |
| Flinterman et al.  2007 [32] | Outcome measured was only a surrogate marker (skin prick test, which determines sensitization but not tolerance) |
| Martınez-Canavate et al. 2009 [33] | Population did not include food allergy  Control group was given probiotics  Outcome measured was only surrogate markers (fecal and blood IgE levels, mucosal IgA levels, natural killer cell levels) |
| Muraro A et al. 2012 [34] | Outcome measured was the hypoallergenicity of the extensively hydrolyzed formula with probiotics, not on the effectiveness of probiotics as treatment for food allergy |
| Nowak-Wegrzyn et al. 2015 [35] | Control group was given probiotics  Outcome measured was the hypoallergenicity of probiotic-containing whey formula compared to probiotic-containing casein formula |
| Pohjavuori et al. 2004 [36] | Outcome measured was only a surrogate marker (interferon levels) |
| Salmi et al. 2010 [37] | Outcome measured was only a surrogate marker (urinary organic acid concentration) |
| Scalabrin et al. 2017 [38] | Population included healthy infants with no food allergy |
| Szajewska et al. 2007 [39] | Population included infants with rectal bleeding, regardless of etiology |
| Tang et al. 2015 [40] | Intervention included probiotic administration with immunotherapy (co-intervention) |
| Viljanen et al. 2005 [41] | Outcome measured was only surrogate markers (Fecal IgA, tumor necrosis factor, antitrypsin, eosinophilic cationic protein levels) |
